# Repurposing of Empagliflozin as a Possible Treatment for Neutropenia and Inflammatory Bowel Disease in Glycogen Storage Disease Type Ib: A Case Report

**DOI:** 10.7759/cureus.27264

**Published:** 2022-07-25

**Authors:** Konstantinos Makrilakis, Aikaterini Barmpagianni, Maria Veiga-da-Cunha

**Affiliations:** 1 First Department of Propaedeutic Internal Medicine, Laiko General Hospital, National and Kapodistrian University of Athens School of Medicine, Athens, GRC; 2 Academic Research, de Duve Institute, Université catholique de Louvain (UCLouvain), Brussels, BEL

**Keywords:** sglt-2 inhibitor, inflammatory bowel disease, neutropenia, empagliflozin, glycogen storage disease type ib

## Abstract

Glycogen storage disease type Ib (GSD-Ib) is an autosomal-recessive inborn error of carbohydrate metabolism, where severe fasting hypoglycemia is associated (among other manifestations) with neutropenia and neutrophil dysfunction (predisposing to recurrent, potentially life-threatening infections) and inflammatory bowel disease (IBD). Granulocyte colony-stimulating factors (G-CSFs) are commonly used for its treatment. Although they have improved the prognosis of the disease, these medicines have also led to concerns about complications associated with their use (namely splenomegaly and hematopoietic malignancies), not to mention their increased cost. Recently, a novel new treatment for neutropenia associated with this disease was discovered. It was found that sodium-glucose cotransporter type 2 (SGLT-2) inhibitors, usually used for the treatment of diabetes mellitus, can ameliorate both neutropenia and IBD-related symptoms and improve the quality of life in patients suffering from these diseases. They do it by inhibiting the renal reabsorption of 1,5-anhydroglucitol, a dietary analog of glucose, whose accumulation due to the specific enzyme deficiency leads to toxic effects on granulocytes. Herein we report the treatment of an adult patient suffering from GSD-Ib with empagliflozin, an SGLT-2 inhibitor.

## Introduction

Glycogen storage disease type Ib (GSD-Ib) is a rare (1/500,000 live births) autosomal-recessive inborn error of carbohydrate metabolism, caused by biallelic mutations in the SLC37A4 (solute carrier family 37 member 4) gene, which encodes the ubiquitously expressed microsomal glucose-6-phosphate transporter (G6PT) enzyme in the endoplasmic reticulum (ER) membrane, resulting in its deficiency. In the liver and kidneys, this enzyme translocates glucose-6-phosphate (G6P) from the cytoplasm into the ER, where it is hydrolyzed to glucose for energy production [1]. Consequently, GSD-Ib usually presents in childhood and is characterized by hypoglycemia, excessive glycogen accumulation in the liver and kidneys, abnormal metabolic serum profiles (namely hyperlipidemia, hyperuricemia, hyperlactatemia), hepatomegaly, anemia, and renal dysfunction [2]. Furthermore, neutropenia and myeloid dysfunction (with a propensity to various, potentially life-threatening recurrent infections) [3], and inflammatory bowel disease (IBD), are characteristics of the condition [4]. Neutropenia frequently necessitates treatment with a granulocyte colony-stimulating factor (G-CSF), a costly and potentially dangerous regimen for neoplastic development, splenomegaly, and osteoporosis [5].

Recently, the pathophysiology of neutrophil dysfunction in these patients was elucidated [6]. It was shown that 1,5-anhydroglucitol-6-phosphate (1,5-AG6P), a non-canonical metabolite resulting from a side reaction of hexokinases on 1,5-anhydroglucitol (1,5-AG), an abundant polyol present in the blood, accumulates within granulocytes. 1,5-AG6P is a potent inhibitor of hexokinases (which catalyze the first step of glycolysis). Its accumulation leads to the energy deficit and subsequent apoptosis of neutrophils in GSD-Ib patients (and in patients with mutations in the glucose-6-phosphatase catalytic subunit 3 [G6PC3] gene, which encodes the catalytic subunit of glucose-6-phosphatase-β, leading to a disease called “severe congenital neutropenia”) [1]. Normally, 1,5-AG6P is transported by G6PT into the endoplasmic reticulum (ER) of granulocytes, where it is dephosphorylated by the phosphatase G6PC3. Furthermore, it was shown that treatment with a renal sodium-glucose cotransporter type 2 (SGLT-2) inhibitor, a class of medications used for treating diabetes mellitus, increased the urinary excretion of 1,5-AG and lowered its concentration in the blood. This improved neutropenia, neutrophil dysfunction, and IBD-related symptoms in these patients [7-12].

Herein we present the case of an adult patient with genetically confirmed GSD-Ib. Her neutropenia and IBD-related symptoms were successfully treated with empagliflozin, an SGLT-2 inhibitor.

## Case presentation

A 32-year-old woman with a history of genetically confirmed GSD-Ib since birth (compound heterozygote for the p.(Gly19Arg), c.55G>C mutation in exon 2 and the p.(Leu348fs) c.1042_1043del mutation in exon 8 of the SLC37A4 gene) was being followed in our clinic. She had had frequent infections (>120 in her lifetime), in multiple sites, namely aphthous stomatitis, periodontitis, otitis, cases of pneumonia, upper respiratory, skin, and urogenital infections, which had necessitated frequent hospital admissions for antibiotic treatment. She was being treated with daily subcutaneous G-CSF injections (filgrastim 30 million units), allopurinol (for hyperuricemia), mesalazine (for IBD), and denosumab (60 mg every six months) for osteoporosis. Her absolute neutrophil count (ANC) ranged from around 200 to 1000/μl. She also reported frequent stools with watery-soft consistency and nearly daily abdominal pains. Her adult Crohn’s Disease Activity Index (CDAI) [13] was 356 (>150 depicts active colitis).

After informed consent, she was started on empagliflozin; initially, 10 mg/day, increasing after two weeks to 10 mg twice a day and after one month to 25 mg/day (corresponding to 0.4 mg/kg/day). Intermittently scanned flash-glucose monitoring during the first weeks of treatment with empagliflozin did not reveal any increased hypoglycemias. The effects of therapy on neutropenia and IBD were impressive. The frequency of infections decreased substantially after starting empagliflozin, to nearly absent. Her ANC fluctuated from around 700 to 1600/μl, and her G-CSF injections were decreased to every four days and gradually to once weekly. The hemoglobin/hematocrit levels also improved (Table 1). Her bowel movements normalized, without diarrheas or abdominal pains, and she gained 6 kgs in 10 months. She was also able to stop mesalazine. The CDAI decreased to 52, and stool calprotectin levels decreased from 141 to 35 μg/g (>50 shows intestinal inflammation) [14]. In addition, 1,5-AG levels in the blood decreased from 140 to 34 μM after ten months of treatment (Table 1). The patient’s quality of life improved dramatically. She could wear earrings for the first time in her life without ear infections. Also, there were no urinary tract or genital mycotic infections, the most frequent side effects of these medications.

**Table 1:** Laboratory findings and inflammatory bowel disease score before and 10 months after empagliflozin treatment. ANC: Absolute neutrophil count; CDAI: Crohn’s Disease Activity Index; 1,5-AG: 1,5-anhydroglucitol.

Variable	Reference range	Before empagliflozin	After empagliflozin
WBC (per μl)	4500-11000	1610	2600
ANC (per μl)	1500-6600	700	1700
Hemoglobin (g/dl)	12.0-16.0	11.9	13.3
Hematocrit (%)	38.0-47.0	35.7	40.7
Uric acid (mg/dl)	2.4-6.0	5.4	6.0
Triglycerides (mg/dl)	50-150	138	149
CDAI	<150	356	52
Stool calprotectin (mg/g)	<50	141	35
Serum 1,5-AG (μΜ)	12-29	140	34

## Discussion

We describe the impressive treatment of neutropenia and IBD-related symptoms in an adult patient with GSD-Ib. We confirm that empagliflozin is a new alternative treatment for these disease manifestations. Moreover, it is superior, cheaper, and safer than G-CSF injections, which have been the mainstay of neutropenia treatment so far. Empagliflozin acts by inducing glucosuria, which increases urinary excretion of 1,5-AG, decreases its availability in the blood and its toxic impact on the energy balance required for granulocytes to survive and function [7].

1,5-AG (also called 1-deoxyglucose) is a structural dietary analog of glucose with an unknown physiologic role. It originates primarily from dietary sources and has a constant concentration in the blood in physiologic conditions, which is adjusted by urinary excretion in the kidneys [15,16]. Most 1,5-AG filtered in the glomeruli is reabsorbed at a specific fructose-mannose active transporter in the renal tubules [17], and this reabsorption is competitively inhibited by glucose. Thus, increased glucosuria (as occurs in uncontrolled diabetes mellitus or with the use of SGLT-2 inhibitors) increases urinary 1,5-AG excretion and decreases its blood concentrations [15].

1,5-AG in the blood is transported into neutrophils and phosphorylated to form 1,5-AG6P, after which G6PT transports it into the ER, where it is dephosphorylated by glucose-6-phosphatase-β, and thus inactivated. In GSD-Ib patients, however, the intracellular accumulation of 1,5-AG6P in the neutrophils, due to deficiency of G6PT, leads to inhibition of hexokinases, the enzymes that catalyze the first step of glycolysis. Their intracellular accumulation may therefore block glucose utilization, explaining neutropenia and neutrophil dysfunction [18]. Unlike the standard G-CSF treatment, which does not directly address the cause of neutropenia, SGLT-2 inhibitors actively eliminate 1,5-AG from the blood (and 1,5-AG6P from granulocytes), explaining the remarkable clinical improvement seen in our patient [6], representing the most significant advancement seen in this field in decades [19]. Quite a few studies have shown this beneficial clinical effect of empagliflozin treatment [7-12]. Like our patient, the ANC count of GSD-Ib patients treated with empagliflozin either increase in numbers or stabilize with treatment. Still, the most significant effect is seen clinically, with elimination (or considerable reduction) in the frequency of infections, due to functional improvement of leukocytes’ actions. This leads to a substantial improvement in their quality of life [7]. Our patient stopped having any infections entirely for the whole ten months of treatment observation (with simultaneously substantially decreasing subcutaneous G-CSF injections) and was able to wear earrings for the first time in her life without an infection.

Another significant improvement in GSD-1B patients’ quality of life with empagliflozin treatment is the IBD-related symptoms. Our patient’s daily abdominal pains were eliminated, and the bowel consistency also improved, leading to weight gain, most likely due to improved intestinal health. She was also able to stop mesalazine at the same time, decreasing her financial and psychological burden. This clinical improvement was also accompanied by significant improvement in her adult CDAI, which indicates bowel health, although not specific to GSD-Ib colitis [13]. The mechanism of the IBD-related disease improvement with empagliflozin is not well elucidated yet. Still, as others have already seen [9,20], better functioning leukocytes, in general, will likely positively impact the inflammatory pathways of IBD pathophysiology.

Treatment with empagliflozin also improved the patient’s anemia, corroborating the results of previous studies [7,12]. The mechanism of anemia in GSD-Ib is multifactorial. It involves the restricted nature of the diet, chronic lactic acidosis, renal involvement, bleeding diathesis, chronic nature of the illness, suboptimal metabolic control, hepatic adenomas, and irritable bowel disease [2]. The improvement with empagliflozin treatment has been postulated to be associated with better intestinal iron absorption due to improved bowel health [12]. However, iron and ferritin levels in our patient were not different before and after initiation of empagliflozin.

It is worth noting that our patient did not develop hypoglycemic episodes while on empagliflozin treatment, which is one of the most feared side effects of this treatment (given that GSD-I predisposes to hypoglycemias anyway). Hypoglycemia has been reported in both pediatric and adult GSD-Ib patients treated with empagliflozin [12,20], although it may be numerically a bit more frequent in children [20]. This issue needs further investigation in future studies since empagliflozin is not officially approved for treating diabetes mellitus in children. In addition, there is not much experience with its proper doses and formulations.

## Conclusions

GSD-Ib is a rare, potentially life-threatening disease of carbohydrate metabolism, for which neutropenia/neutrophil dysfunction and IBD have, up to recently, been causing reduced quality of life of affected persons. Neutropenia has been traditionally treated with subcutaneous injections of G-CSF. Although these injections have improved the prognosis of the disease, they have also led to concerns about complications associated with their use (splenomegaly and hematopoietic malignancies) and increased cost. Furthermore, IBD is not substantially affected by this treatment. We have shown that empagliflozin is an effective treatment of neutropenia/ neutrophil dysfunction and IBD-related symptoms in an adult patient with GSD-Ib, by reducing 1,5-AG in the blood without causing hypoglycemia.
